# Distribution, Polymorphism and Function Characteristics of the GST-Encoding *Fhb7* in *Triticeae*

**DOI:** 10.3390/plants11162074

**Published:** 2022-08-09

**Authors:** Xianrui Guo, Mian Wang, Houyang Kang, Yonghong Zhou, Fangpu Han

**Affiliations:** 1 State Key Laboratory of Plant Cell and Chromosome Engineering, Institute of Genetics and Developmental Biology, Innovation Academy for Seed Design, Chinese Academy of Sciences, Beijing 100101, China; 2 University of Chinese Academy of Sciences, Beijing 100049, China; 3 Triticeae Research Institute, Sichuan Agricultural University, Chengdu 611130, China

**Keywords:** horizontal gene transfer, *Fhb7*, *Triticeae*, *Thinopyrum*, Fusarium head blight

## Abstract

Encoding a glutathione S-transferase (GST) and conferring resistance to Fusarium head blight (FHB), *Fhb7* was successfully isolated from the newly assembled *Thinopyrum elongatum* genome by researchers, with blasting searches revealing that *Thinopyrum* gained *Fhb7* through horizontal gene transfer from an endophytic *Epichloë* species. On the contrary, our molecular evidence reveals that the homologs of *Fhb7* are distributed commonly in *Triticeae*. Other than *Thinopyrum*, the *Fhb7* homologs were also detected in four other genera, *Elymus*, *Leymus*, *Roegneria* and *Pseudoroegneria*, respectively. Sequence comparisons revealed that the protein sequences were at least 94% identical across all of the *Fhb7* homologs in *Triticeae* plants, which in turn suggested that the horizontal gene transfer of the *Fhb7* might have occurred before *Triticeae* differentiation instead of *Thinopyrum*. The multiple *Fhb7* homologs detected in some *Triticeae* accessions and wheat-*Thinopyrum* derivatives might be attributed to the alloploid nature and gene duplication during evolution. In addition, we discovered that some wheat-*Thinopyrum* derivatives carrying the *Fhb7* homologs had a completely different reaction to Fusarium head blight, which made us question the ability of the GST-encoding *Fhb7* to resist FHB.

## 1. Introduction

It is reported that beneficial plant-associated microbe and fungi possess widespread effects on plant growth and defense throughout their evolutionary history [1]. Researchers discovered that microbes influenced plant responses to global changes through at least four mechanisms: physical modification of the environment, secreting chemicals that mimic plant hormones, altering plant gene expression, and facilitating plant nutrient acquisition [2]. For example, in plants, bacteria in the genus *Azospirillum* have the capability of fixing nitrogen in the soil [3]. Other examples can be found in endophytes, organisms that inhabits the internal tissues of a plant without resulting in visible disease symptoms [4]. These include potentially beneficial micro-organisms for the plant, such as the endophytic fungal *Colletotrichum tropicale*, which induces the expression of hundreds of host defense-related genes in *Theobroma cacao* and resulting in stronger pathogen resistance [5].

Horizontal gene transfer (HGT) is the transmission of genetic material across the genome of biological organisms with reproductive barriers, which are particularly common in endophytic fungi species and their hosts [6,7]. On the one hand, HGT can facilitate microbial adaptation to adverse conditions in the environment and inside the host plant. The ciliate *Tetrahymena thermophile* acquired the genes involved in the catabolism of complex carbohydrates from bacteria and archaea, and these genes facilitated the Ciliates’ colonization of the rumen [8]. Eighteen *Rhizophagus irregularis* genes were found to be recently acquired from either plants or bacteria and these acquired genes may participate in diverse but fundamental biological processes such as regulation of gene expression, mitosis, and signal transduction [9]. On the other hand, the horizontal gene transfer and resultant integration of the transferred material might provide the host with adaptive advantages towards environment changes and acquisition of new traits and functions [7]. A study reported that a gene encoding β-1, 6-glucanase was transferred from fungal endophyte to a cool-season grass host and may function to protect against infection by other fungal pathogens [10]. Another example is that 128 genes of 57 families in the moss *Physcomitrella patens* were identified as derived from prokaryotes, fungi, or viruses. These acquired genes are involved in essential or plants specific activities such as xylem formation, plant defense, and nitrogen recycling, as well as the biosynthesis of starch, polyamines, hormones and glutathione, which played critical roles in the transition of plants from aquatic to terrestrial environments [11].

Different methods have been proposed to identify HGT events, such as relying on gene distribution patterns, unexpected phylogenetic tree topology, and similarity search between genomes [7,12]. The genome composition, including the base compositions, the patterns of codon usage, and the frequencies of di- and trinucleotides in DNA sequence, were also the clues to identifying HGT events [7,13]. *Triticeae*, one tribe of the grasses, comprises many economical important foods (wheat, barley, and rye) and some fine forage accessions, including the genera *Thinopyrum*. Previous reports indicate that endophytic *Epichloë* species were discovered among many grass genera in *Triticeae*, such as *Elymus*, *Leymus*, *Roegneria* and *Agropyron* [14]. With the increasing availability of genomic data, more and more horizontal gene transfer events from microbe to plants are being identified.

Fusarium head blight (FHB), mainly caused by the fungus *Fusarium graminearum*, is currently one of the most economically important wheat diseases in the world, which not only results in grain yield loses but also reduces grain quality due to mycotoxin contamination [15]. *Fusarium* species easily cloned on the head at the flowering stage and subsequent infection bleached the wheat spikes and shrank the kernels. FHB resistance has been controlled quantitatively and more than 432 quantitative trait loci (QTLs) have been mapped on all wheat chromosomes by linkage mapping or by association mapping [16]. Among these QTLs, researchers have formally named seven FHB-resistant ones: *Fhb1* on chromosome 3BS, *Fhb2* on chromosome 6BS, *Fhb3* introgressed to chromosome 7AS from *Leymus racemosus* chromosome 7Lr#1, *Fhb4* on chromosome 4BL, *Fhb5* on chromosome 5AS, *Fhb6* on chromosome 1A from 1Ets#1S of *Elymus tsukushiensis* and *Fhb7* on chromosome 7E2 from *Thinopyrum ponticum* [17,18,19,20,21,22,23]. Using recombinant inbred lines derived from a cross between two Thatcher-*Th. ponticum* substitution lines, K11463 (7E1/7D) and K2620 (7E2/7D), the major FHB resistance locus *FhbLoP* (designated as *Fhb7* now) was mapped to the very distal region of the long arm of chromosome 7E2 [23,24]. Wang et al. sequenced the genome of *Th. elongatum* (D-3458) and successfully mapped and cloned *Fhb7*, which encodes a glutathione S-transferase and confers wheat broad resistance to Fusarium head blight [25]. By blasting against the National Center for Biotechnology Information (NCBI) GenBank database, the authors did not find any homolog of *Fhb7* in the *Triticum* genus or in the entire plant kingdom. They found that the *Fhb7* homologs were distributed among *Epichloë* species, endophytic fungi of temperate grasses. Phylogenetic analyses suggested the *Fhb7* in *Thinopyrum* wheatgrass was transferred from *Epichloë* species through horizontal gene transfer [25]. Based on these results, we were curious about why *Fhb7* was transferred from *Epichloë* species only to *Thinopyrum* and whether there was a special mechanism for the transfer from *Epichloë* species to *Thinopyrum*.

In recent years, many genomes of *Triticeae* have been deciphered, including diploid, tetraploid, hexaploid wheat species, barley, and rye. However, due to lack of detailed genomic data for many other species in *Triticeae*, a single blast search may not accurately characterize the *Fhb7* distribution in the entire plant kingdom. In this study, we checked the *Fhb7* distribution among *Triticeae* by using specific molecular markers. Our results demonstrate that *Fhb7* homologs are in fact distributed commonly in *Triticeae* and the horizontal gene transfer may have occurred before *Triticeae* differentiation instead of being unique to *Thinopyrum*.

## 2. Materials and Methods

### 2.1. Materials

The wheat-*Th. elongatum* addition line CS-7EL, the wheat-*Th. elongatum* substitution line 7E/7D and the common wheat Chinese Spring (CS) were provided by Mingcheng Luo (UC Davis Plant Sciences). The wheat-*Th. ponticum* partial amphiploids XY693 and XY784 were provided by Zhensheng Li (Institute of Genetics and Developmental Biology, Chinese Academy of Sciences). The wheat-*Th. ponticum* partial amphiploid SNTE122 was provided by Honggang Wang (Shandong Agricultural University) and the wheat-*Th. ponticum* translocation lines TNT-B provided by George Fedak (Plant Research Centre, Agriculture Canada, Ottawa, ON, Canada). Moshe Feldman (Weizmann Institute of Sciences) provided the *Th. elonatum* accessions Ae31 and Ae56. Other accessions in *Triticeae* (Table S1) were provided by Yiwen Li (Institute of Genetics and Developmental Biology, Chinese Academy of Sciences).

### 2.2. Methods

#### 2.2.1. Sequence Amplification and Cloning of *Fhb7* Homologs

A 3846-bp length sequence containing the *Fhb7* coding region and its upstream 3 kb sequence were extracted from the released *Th. elongatum* (D-3458) genome. Primers that are specific for coding region and promoter region were designed by using the software Primer5 (Table 1). The primers 26102F and 26102R were used to amplify the coding region of *Fhb7* homologs. The primers 26102ProF and 26102R were used to amplify the promoter and coding regions of *Fhb7* homologs. About ten seeds for all accessions used in this study were germinated on moist filter paper in Petri dishes at room temperature. After five days, three to five seedlings for each accession were sampled for DNA extraction. The genomic DNA was extracted using the cetyltrimethylammonium bromide (CTAB) method. PCR was conducted in a 50 μL reaction volume containing 1 μL 100 ng/μL genomic DNA, 1 μL 10 μmol/L of each primer, 25 μL 2× KOD One™ PCR Master Mix (Toyobo Biotech Co., Ltd., Osaka, Japan) and 22 μL sterilized ddH_2_O. Amplified PCR products were separated on 1.5% agarose gels at 150 V for 20 min, stained with ethidium bromide and visualized using ultraviolet (UV) light. The amplified DNA product was purified by Universal DNA Purification Kit (Tiangen Biotech Co., Ltd., Beijing, China) and cloned into the *pEASY^®^*-Blunt Simple Cloning Vector (TransGen Biotech Co., Ltd., Beijing, China).

#### 2.2.2. Chromosome Preparation and Fluorescence In Situ Hybridization (FISH)

The seeds of the line CS-7EL, 7E/7D, SNTE122, and TNT-B were germinated on moist filter paper in a petri dish at room temperature for two to three days. The main roots were cut from the seedlings and placed in nitrous oxide for 2 h. Subsequently the roots were fixed in 90% acetic acid for 5 min and then washed three times by using sterile water. The section containing dividing cells was cut and digested in 20 μL 1% pectolyase Y23 and 2% cellulase Onozuka R-10 solution for 1 h at 37 °C. After digestion, the root sections were washed in 75% ethanol two times briefly. The root sections were carefully broken by using a needle and collected by centrifugation. The sedimentation was resuspended in 100% acetic acid solution. The cell suspension was dropped onto glass slides in a wet box and dried slowly.

The probes were labeled using the nick translation method. 7EL-1 was obtained by Dop-PCR from the 7EL library constructed by chromosome microdissection. It was specific for the genome of *Th. elongatum* and *Th. ponticum*. The centromeric retrotransposon of wheat (CRW) clone 6C6 was labelled with Texas-red-5-dCTP. The 2846-bp *Fhb7* homolog in the line CS-7EL and the probe 7EL-1 were labeled with Alexa Fluor-488-5-dUTP. The labeled probes were dissolved in 2 × SSC and 1× TE, dropped to the chromosome spreads. The slides were covered with a plastic sheet and denatured by heating at 100 °C for 5 min. After denaturing, the slides were placed into a moisturizing aluminum box with a lid and transferred into an incubator held at 55 °C overnight for hybridization. Then the slides were washed in 2× SSC buffer and the chromosomes were stained with DAPI (4′,6-diamidino-2-phenylindole).

#### 2.2.3. FHB Resistance Evaluation on Wheat-Thinopyrum Derivatives

The FHB resistance evaluation was performed in field condition in Beijing (116°42′ E, 40°10′ N). To keep the flowering dates close, all plants were sown in stages. *F. graminearum* strains (Fg16-2, Fg16-5 and Fg16-11) and *Fusarium asiaticum* strain (Fa301) in Mung bean were mixed and 20 μL fungal suspension (1 × 10^6^ conidia/mL) was injected into the central spikelet at early flowering stage. The wheat cultivar Jimai 22 was used as the susceptible control. For each of the lines, at least 10 spikes were inoculated with *Fusarium* species and the inoculated spikes were covered with a plastic bag for 2 days to keep moist for fungal infection. The number of diseased spikelets for each spike was recorded at 7, 14, and 21 days after inoculation. The statistical analysis was performed by the unpaired t test using the software GraphPad Prism 8.

#### 2.2.4. The Expression Analysis of the *Fhb7* Homolog in the Line TNT-B

Three spikelets around the inoculated one from at least three spikes from different plants of the line TNT-B were collected at 96 h post inoculation and grounded in liquid nitrogen for total RNA extraction using TRIzol^®^ Reagent (Thermo Fisher Scientific lnc., Shanghai, China). First-strand cDNA synthesis from the total RNA was performed by using the FastKing RT kit (with gDNase) (TianGen Biotech Co., Beijing, China). The expression analyses were performed using the primers 26102RT-F and 26102RT-R (Table 1). The gene *actin* was used as an internal standard by the primers Actin-F and Actin-R. The relative expression of the *Fhb7* homolog was calculated by the 2^−ΔΔCT^ method.

## 3. Results

### 3.1. Fhb7 Is Not Unique to Thinopyrum in Triticeae

**Figure 1:**
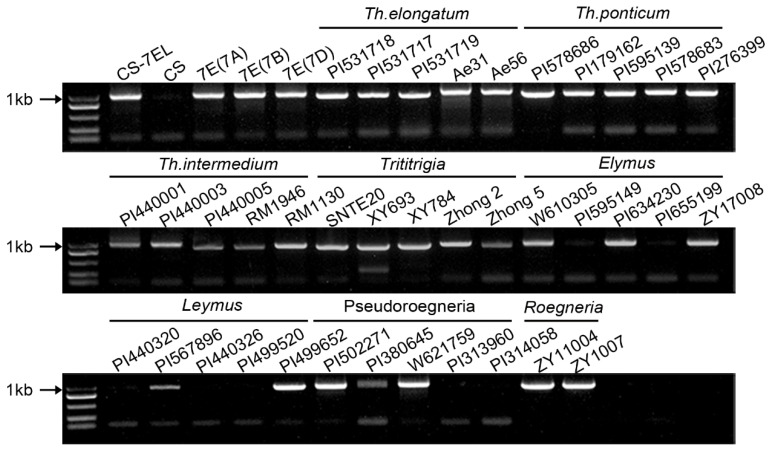
Detection of the *Fhb7* homologs among different species in *Triticeae*. *Th. elongatum*, diploid, 2n = 2x = 14. *Th.ponticum*, decaploid, 2n = 10x = 70. *Th. intermedium*, hexaploid, 2n = 6x = 42. *Trititrigia* means partial amphiploid produced from distant hybridization between common wheat and *Thinopyrum. Elymus*, *Leymus*, *Pseudoregneria,* and *Roegneria* represent four genera in *Triticeae*.

We collected 126 different accessions belonging to *Triticeae* to check the *Fhb7* distribution via polymerase chain reaction (Table S1). *Fhb7* homologs were indeed detected in the genera *Thinopyrum*, including *Th. elongatum* (2n = 2x = 14), *Th. ponticum* (2n = 10x = 70) and *Th. intermedium* (2n = 6x = 42) (Figure 1). These results were confirmed by detecting the *Fhb7* homologs in some artificially synthesized wheat-*Thinopyrum* derivatives, such as the wheat-*Th. elongatum* addition line CS-7EL, wheat-*Th. ponticum* partial amphiploid XY693 and wheat-*Th. intermedium* partial amphiploid Zhong 2. However, other than the genera *Thinopyrum*, *Fhb7* homologs were also discovered in some accessions among the genera *Elymus*, *Leymus*, *Roegneria* and *Pseudoroegneria*. What is more, unlike *Thinopyrum*, the other four genera all had some accessions that could not detect *Fhb7* homologs, such as the *Elymus* accession PI 655199 and the *Leymus* accession PI 440326.

### 3.2. Polymorphism of Fhb7 Homologs in Triticeae

**Figure 2:**
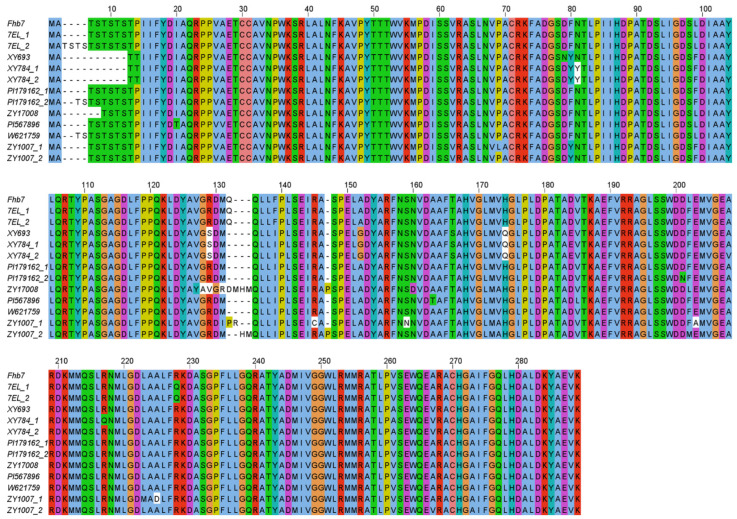
Protein sequence alignments of the *Fhb7* homologs in *Triticeae*. “_1” and “_2” means different *Fhb7* homologs detected in one accession.

**Figure 3:**
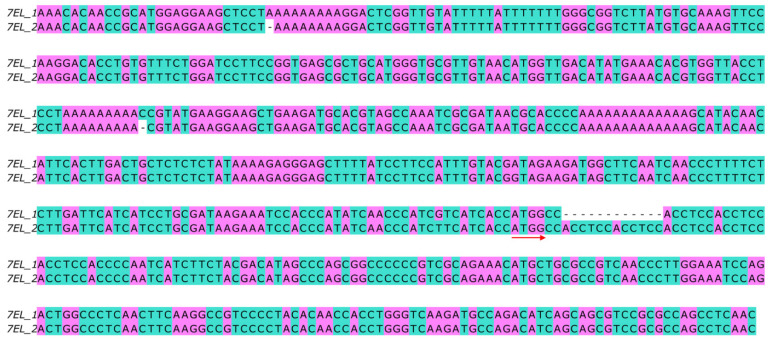
Partial sequence comparison of two *Fhb7* homologs coding region and promoter region in wheat-*Th. elongatum* addition line CS-7EL. The red arrow indicates the initiation codon.

**Figure 4:**
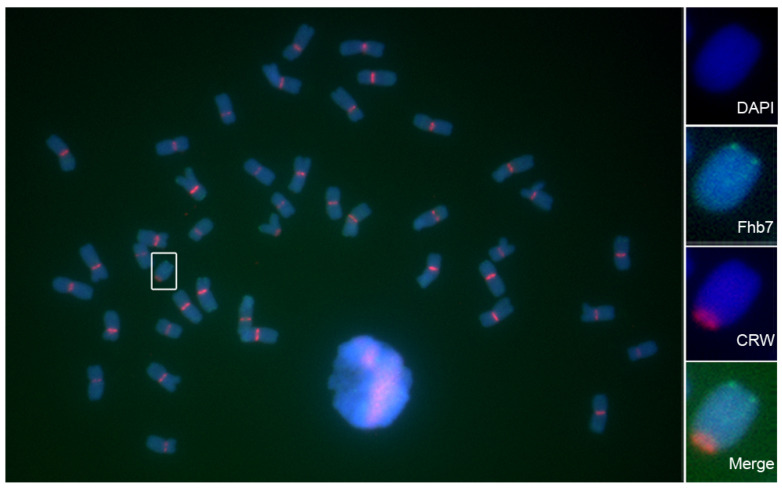
*Fhb7* homolog distribution detected by fluorescence in situ hybridization in the line CS-7EL. The CRW is labeled in red. The 2846-bp *Fhb7* homolog is labeled in green. DAPI staining is labeled in blue. The insets show high-magnification images of chromosomes 7EL in the white box.

*Fhb7* homologs were amplified from different accessions and inserted into the sequenced vector. Sequence comparisons revealed that the protein sequences were at least 94% identical across all of the *Fhb7* homologs in *Triticeae* plants (Figure 2). Despite indel variation and amino acid substitution across all of the homologs of *Fhb7*, no premature termination and code-shifting mutations occurred in the protein sequences. The main variation was the number of Thr-Ser at the amino terminus of the protein sequence (Figure 2). We also noticed that more than one *Fhb7* homologs were detected in some accessions, such as *Th. ponticum* PI 179162 and *Roegneria kamoji* ZY1007. These results were confirmed by detecting more than one *Fhb7* homologs in some artificially synthetic wheat-*Thinopyrum* derivatives, such as the wheat-*Th. elongatum* addition line CS-7EL and the wheat-*Th. ponticum* partial amphiploid XY784. To exclude the possibility of endophytic *Epichloë* species contamination, we used the reference genome of *Th. elongatum* (D-3458) to design primers and tried to amplify the fragment that included *Fhb7* homolog and its partial promoter region. Two similar fragments including a 2 kb promoter were isolated and sequenced. Further analysis revealed that the two sequences were corresponding to the two *Fhb7* homologs detected in CS-7EL, respectively (Figure 3). These results were also confirmed by detecting two *Fhb7* homologs by performing reverse transcription polymerase chain reaction in CS-7EL. To further analyze the distribution characterization of the two *Fhb7* homologs in CS-7EL, fluorescence in situ hybridization was conducted on the metaphase chromosomes in CS-7EL by using a 2846-bp probe including the complete coding region of the *Fhb7* homolog and its 2 kb promoter. It was obvious that only one signal was detected at the end of the alien chromosome 7EL (Figure 4). These results suggested that all *Fhb7* homologs distributed proximal to the chromosome 7EL and each homolog might arrange in close proximity.

### 3.3. Contrast Reactions of Wheat-Thinopyrum Derivatives Carrying Fhb7 Homologs to FHB

**Figure 5:**
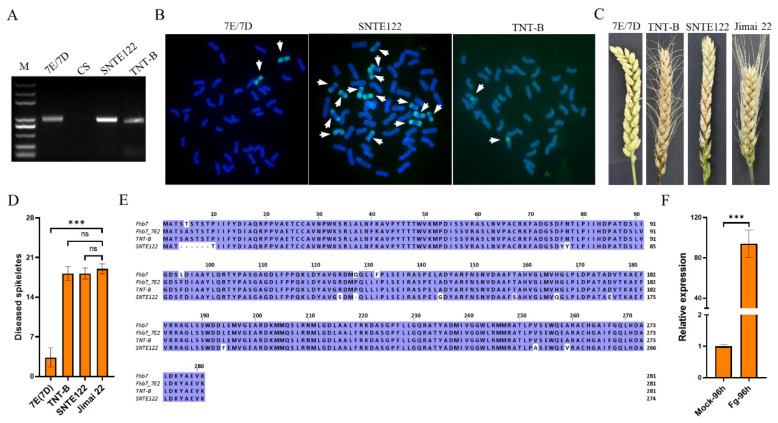
FHB resistance evaluation on wheat-*Thinopyrum* derivatives carrying *Fhb7* homologs. (**A**) Detecting *Fhb7* homolog in wheat-*Thinopyrum* derivatives. (**B**) Karyotype analysis on wheat-*Thinopyrum* derivatives. The white arrows indicate the alien chromosome. (**C**,**D**) FHB resistance evaluation on wheat-*Thinopyrum* derivatives. The spike pictures (**C**) were photographed and the number of diseased spikelets (**D**) were recorded 14 d after inoculation with *Fusarium* species. (**E**) Protein sequence comparison of the *Fhb7* homologs among wheat-*Thinopyrum* derivatives. (**F**) Expression analysis of the *Fhb7* homolog in the line TNT-B. Fg indicated *Fusarium* species. *** *p* < 0.001, ns, *p* > 0.05.

As mentioned above, the *Fhb7* homologs were detected in some artificially synthesized wheat-*Thinopyrum* derivatives. We detected *Fhb7* homologs in wheat-*Th. elongatum* substitution lines 7E/7D, wheat-*Th. ponticum* amphiploid SNTE122 and wheat-*Th. ponticum* translocation line TNT-B (Figure 5A,B). However, we found they have different reactions to FHB: the line 7E/7D showed high level resistance to FHB with only several spikelets bleached while SNTE122 and TNT-B were highly susceptible to FHB with nearly whole spike bleached (Figure 5C,D). To exclude the possibility of sequence variation, we cloned and sequenced the *Fhb7* homologs in them. Compared with *Fhb7*, amino acid substitutions and deletions were discovered in the homolog of the line SNTE122 and only substitutions in that of the line TNT-B (Figure 5E). We also discovered that the protein sequence of the *Fhb7* homolog in TNT-B is the same with that of the homolog in the wheat-*Th. ponticum* substitution line 7E2/7D (Figure 5E), which was used as the resistant parent in the *Fhb7* mapping population [25]. Due to the contrast reaction to FHB, the expression pattern of the *Fhb7* homolog in TNT-B was characterized by qRT-PCR. We surprisingly found that the *Fhb7* homolog in TNT-B was dramatically induced 96 h after inoculation with *Fusarium* species (Figure 5F). Therefore, contrast reactions of wheat-*Thinopyrum* derivatives carrying *Fhb7* homologs to FHB were discovered in our study.

## 4. Discussion

Although several methods have been enumerated for identifying potential HGT events, there are some imperfections in each method [7]. Therefore, researchers suggested that one or more of the above-mentioned methods should be used in combination to properly identify identifying HGT events [7]. Although no *Fhb7* homolog was discovered by blast searching the NCBI GenBank database, it may be inaccurate to draw the conclusion that *Thinopyrum* gained *Fhb7* through the HGT from an endophytic *Epichloë* species [25,26]. Lacking detailed genomic information for many species in *Triticeae*, the true distribution of *Fhb7* homologs in *Triticeae* was masked. In our study, the molecular evidence clearly shows that *Fhb7* homologs are distributed commonly in *Triticeae* and is not exclusive to the genus *Thinopyrum* in the plant kingdom. Our results suggested that the HGT of the *Fhb7* was not an accidental happening by chance only in the genera *Thinopyrum* but may have instead occurred before *Triticeae* differentiation. We wonder when *Triticeae* might have borrowed such an alien gene from *Epichloë* species during evolution. More puzzling is why horizontal gene transfer did not occur to the genera *Triticum* that are known to be seriously affected by *Fusarium* species.

As wheat relative species, *Th. elongatum* and *Th. ponticum* are important reservoirs of elite genes for wheat improvement and the genes for FHB resistance derived from them have been located on the homologous group seven, 7EL for *Th. elongatum* and 7E2 for *Th. ponticum*, respectively [23,27,28]. Although two genes were both located to the homologous group seven, no molecular evidence could verify the relationship between different genes or homologs. Referring to the newly assembled *Th. elongatum* (D-3458) genome, a glutathione S-transferase was identified as a candidate for *Fhb7* by map-based cloning and conferred broad resistance to *Fusarium* species by detoxifying trichothecenes through de-epoxidation [25]. Unlike the results published, we firstly discovered that some wheat-*Thinopyrum* derivatives carrying *Fhb7* homologs were highly susceptible to FHB. For the *Fhb7* homolog in the line SNTE122, the similar protein sequence shared with that derived from the wheat-*Thinopyrum* substitution line 7E2/7D and the induction by *Fusarium* species lead us to suspect the function of *Fhb7* on FHB resistance. Secondly, *Fhb7* was proved by a single copy in the assembled *Th. elongatum* genome [25]. However, more than one *Fhb7* homolog was detected in some *Thinopyrum* accessions and wheat-*Thinopyrum* derivatives in our study. By amplifying the promoter sequence of *Fhb7* homolog, we excluded the possibility of contamination by the endophytic *Epichloë* species. The two expressed *Fhb7* homologs in CS-7EL might be attributed to a recent burst of gene duplications in *Triticeae* [29]. The *Fhb7* in an FHB-resistant substitution line 7E2/7D used for mapping was demonstrated to be semidominant and it was applied into wheat breeding by creating wheat-*Th. ponticum* translocation lines [25]. As wheat-alien chromosomes addition or substitution lines usually carried linkage drags between useful and undesirable genes, translocation lines were preferred to transfer alien genes to common wheat by breeders for its higher possibility of breaking linkage drag [30,31]. Based on the dosage effect of *Fhb7* homolog, the translocation lines derived from the addition line CS-7EL may have better resistance to FHB than the substitution line 7E2/7D. Other than gene duplications, the alloploid nature might be another reason for the absence of more than one *Fhb7* homologs in some accessions, such as *Th. ponticum* PI 179162 and *R. kamoji* ZY1007.

## Figures and Tables

**Table 1:** Primers used in this study.

Primer Name	Sequence (5′-3′)	Tm (°C)	Amplified Region
26102F	CGATAGAAGATAGCTTCAATCAACCCTTT	60	CDS
26102R	CTACTTCACCTCGGCATACTTGTC		
26102ProF	TCCGCATTTCCCTTGCAGAT	60	Promoter and CDS
26102RT-F	GGACTTCCCTTGGATCCTGC	60	CDS
26102RT-R	ACCGACAATCATGTCCGCAT		
Actin-F	CAACGAGCTCCGTGTCGCA	60	CDS
Actin-R	GAGGAAGCGTGTATCCCTCATAG		

CDS indicates the coding region sequence.

## Data Availability

Not applicable.

## Supplementary Materials

The following are available online at https://www.mdpi.com/article/10.3390/plants11162074/s1. Table S1: Distribution of the *Fhb7* homologs in *Triticeae*.
